# What is long-term survival in patients with peritoneal metastasis from gastric, pancreatic, or colorectal cancer? A study of patients treated with systemic chemotherapy and pressurized intraperitoneal aerosol chemotherapy (PIPAC)

**DOI:** 10.1515/pp-2023-0038

**Published:** 2023-12-14

**Authors:** Charlotte G. Kryh-Jensen, Claus W. Fristrup, Alan P. Ainsworth, Sönke Detlefsen, Michael B. Mortensen, Per Pfeiffer, Line S. Tarpgaard, Martin Graversen

**Affiliations:** Odense PIPAC Center (OPC), Odense University Hospital, Odense, Denmark; Upper GI & HPB Section, Department of Surgery, Odense University Hospital, Odense, Denmark; Department of Pathology, Odense University Hospital, Odense, Denmark; Department of Clinical Research, Faculty of Health Sciences, University of Southern Denmark, Odense, Denmark; Department of Oncology, Odense University Hospital, Odense, Denmark; OPEN – Odense Patient Data Explorative Network, Odense University Hospital, Region of Southern Denmark, Odense, Denmark

**Keywords:** long-term survival, peritoneal metastasis, PIPAC, response evaluation, PRGS

## Abstract

**Objectives:**

A definition of long-term survival (LTS) in patients with peritoneal metastasis (PM) from gastric cancer (GC), pancreatic cancer (PC) or colorectal cancer (CRC) treated with systemic chemotherapy and pressurized intraperitoneal aerosol chemotherapy (PIPAC) is lacking. We aimed to define LTS and investigate characteristics and treatment response in patients who reached LTS in data from two prospective trials.

**Methods:**

Retrospective study of patients with GC-, PC-, or CRC-PM from the prospective PIPAC-OPC1 and PIPAC-OPC2 studies. The definition of LTS was based on published systematic reviews and randomized controlled trials. LTS was defined at the time point where 25 % of the patients were alive in these studies. Histology based response was evaluated by the mean Peritoneal Regression Grading Score (PRGS) using biopsies obtained prior to PIPAC 3, and defined by a mean PRGS of ≤2.0 or a decrease of mean PRGS of ≥1, compared to baseline.

**Results:**

LTS was defined at 21 (GC), 15 (PC), and 24 (CRC) months. Fifty-one (47.2 %) patients (nine GC, 17 PC, 25 CRC) reached LTS calculated from the date of PM diagnosis. All but one received palliative chemotherapy before PIPAC, and 37 % received bidirectional treatment. More than 90 % of the LTS patients had response according to PRGS. The mOS from PIPAC 1 was 23.3, 12.4, and 28.5 months for GC, PC, and CRC LTS patients.

**Conclusions:**

Patients with PM from GC, PC, and CRC treated with systemic chemotherapy and PIPAC can reach LTS and most show histological response. Causality must be further investigated.

## Introduction

Peritoneal metastasis (PM) is mostly seen in patients with abdominal cancers. Incidence, treatment option, and prognosis depends on origin and type of primary tumor. Risk factors are not fully understood, but include genetic alterations, nodal status, and serosal involvement of primary tumor [1]. Patients with PM from gastric cancer (GC), pancreatic cancer (PC), or colorectal cancer (CRC) have a dismal prognosis, and are often troubled by severe symptoms such as ascites, fatigue, pain, and bowel obstruction [2], [3], [4], [5], [6]. Selected patients with CRC-PM are eligible for cytoreductive surgery, but most patients will die from their disease [7, 8]. A systematic review and meta-analysis of randomized controlled trials in patients with metastatic CRC treated with systemic chemotherapy included 14 studies of 10,553 patients [3]. It demonstrated a shorter median overall survival (mOS) in patients with CRC-PM (16.3 months) compared to patients with CRC liver (19.1 months) or lung metastasis (24.6 months). Data from a randomized trial using intraperitoneal chemotherapy in patients with GC-PM suggest mOS up to 18 months, but data on patients with isolated PM from pancreatic cancer are more scarce [9], [10], [11].

Pressurized intraperitoneal aerosol chemotherapy (PIPAC) was introduced a decade ago as a local treatment of PM, where chemotherapeutic agents are nebulized within the peritoneal cavity during laparoscopy [12, 13]. PIPAC is well tolerated and may be completed in an outpatient clinic [14], [15], [16], [17], [18]. Patients with CRC-PM are usually treated with oxaliplatin, and other PM patients with cisplatin and doxorubicin. The efficacy of PIPAC has not been evaluated in randomized trials, and only few prospective studies have been published [14, 19], [20], [21], [22], [23], [24], [25], [26]. Most of these studies are hampered by heterogeneous study populations such as differences in performance status, frequency of metachronous or synchronous PM, and types and lines of systemic chemotherapy before, during, or after PIPAC. Not surprisingly, the mOS after the first PIPAC treatment (PIPAC 1) varies substantially from 4.7 to 15.4 months in GC-PM patients, 6–12.7 months in PC-PM patients, and 9.9–27.0 months in CRC-PM patients [16, 18], [19], [20, 27], [28], [29], [30], [31], [32], [33], [34], [35], [36]. It is currently unclear whether these differences in survival both within and between studies are due to a multimodal treatment strategy including both systemic chemotherapy and PIPAC. It is also not known, which treatment modality may yield long-term survival (LTS) among patients with PM from GC, PC, and CRC, and there is no generally accepted definition of LTS among these entities.

This descriptive study was designed to recommend a definition of LTS in patients with GC-, PC-, and CRC-PM, treated with a combination of systemic chemotherapy and PIPAC. It aimed to identify baseline characteristics, treatment related data, and the histological or cytological response to PIPAC in these patients.

## Materials and methods

This is a retrospective subgroup analysis of all patients with GC-, PC-, or CRC-PM that were included in the prospective PIPAC-OPC1 and PIPAC-OPC2 studies, including 33 patients treated with electrostatic precipitation (ePIPAC) [14, 24, 37].

Patients were enrolled at Odense PIPAC Center (OPC), Odense, Denmark, and discussed at a dedicated PIPAC multi-disciplinary team conference prior to inclusion. The in- and exclusion criteria and technical aspects of the PIPAC procedure have been described previously [14, 24, 37]. Patients were scheduled for a series of three (e)PIPACs at an interval of four to six weeks. The interval was increased to seven weeks if they received concomitant systemic chemotherapy (bidirectional treatment).

### Definition of long-term survival (LTS)

The definition of LTS was defined from the time of PM diagnosis based on a review of randomized controlled trials (RCT) in patients with metastatic GC, PC, or CRC treated with systemic chemotherapy [3, 38], [39], [40], [41]. We used specific large-scale data on PM patients if they were available. Otherwise, we used data from patients with unspecified metastatic disease. Long-term survival was pre-defined as the time point where only 25 % of the patients were alive.

### Evaluation of treatment response

The objective response to previous systemic treatment was assessed using the histological Peritoneal Regression Grading Score (PRGS) in peritoneal quadrant biopsies and with cytologic evaluation of ascites or peritoneal lavage fluid (PLF), both obtained before nebulization of chemotherapy prior to PIPAC 1 [14, 37, 42], [43], [44], [45]. Similarly, the objective response to PIPAC/bidirectional treatment was assessed by PRGS and cytology prior to PIPAC 3. If response data from PIPAC 3 were missing, data from PIPAC 2 were used instead. All PRGS and cytology was assessed by the same pathologist with an interest in peritoneal pathology (SD).

### Definition of treatment response

Response to treatment was defined as a mean PRGS ≤2 at PIPAC 3 or an absolute decrease of mean PRGS ≥1.0 from PIPAC 1 to PIPAC 3, in accord with the PIPAC-OPC2 study [37]. Cytology based response or progression was defined as a change from positive to negative or from negative to positive cytology from PIPAC 1 to PIPAC 3. Positive cytology was defined as malignant cells or cells suspicious of malignancy in ascites/PLF, as described previously [42].

### Statistics

Values were given as means (SD) or medians (range) when appropriate. We performed no comparisons or testing of significance due to the small sample size, and data were analysed by the intention to treat principle. Data collection ended February 1, 2023, and survival data from PIPAC 1 was computed with Kaplan Meier statistics.

### Ethical clearance

The PIPAC-OPC1 and PIPAC-OPC2 study protocols, including the amendment of ePIPAC, were approved by the Regional Committees on Health Research Ethics for Southern Denmark (Project- ID: S-20140211 and S-20160100), the Danish Medicines Agency, and registered at www.clinicaltrials.gov (ClinicalTrials.gov identifier: NCT02320448 and NCT03287375), and the European Clinical Trials Database (EudraCT) number 2016-003394-18.

The studies complied with the Helsinki Declaration. Patients gave oral and written informed consent.

## Results

### Definition of long-term survival

#### Peritoneal metastasis from gastric cancer

Cunningham et al. randomly assigned 1,002 patients with non-resectable or metastatic oesophagogastric cancer to different triplet therapies. The 25 % survival after epirubicin/cisplatin/fluorouracil and epirubicin/oxaliplatin/capecitabine was 16 months and 21 months, respectively [39]. Van Cutsem et al. randomly assigned 445 patients with advanced gastric or oesophagogastric junction adenocarcinoma to triplet or doublet chemotherapy. The 25 % survival after docetaxel/cisplatin/fluorouracil and cisplatin/fluorouracil was 18 months and 15 months, respectively [40]. Based on these studies and the randomized study on the combination of intraperitoneal and systemic chemotherapy by Ishigami et al., we defined LTS for GC-PM patients as survival of at least 21 months [9].

#### Peritoneal metastasis from pancreatic cancer

There are no randomized trials of patients with isolated PC-PM. Von Hoff et al. randomly assigned 861 patients with metastatic PC to doublet or monotherapy. The 25 % survival after nab-paclitaxel/gemcitabine and gemcitabine alone was 15 months and 12 months, respectively [41]. Conroy et al. randomly assigned 342 patients with metastatic PC to FOLFIRINOX (oxaliplatin/irinotecan/leucovorin/fluorouracil) or gemcitabine. The 25 % survival after FOLFIRINOX and gemcitabine was 16 months and 11 months, respectively [38]. Based on these studies, we defined LTS for PC-PM patients as survival of at least 15 months.

#### Peritoneal metastasis from colorectal cancer

The systematic review and meta-analysis by Franko et al. included data from 14 RCTs of 10,533 patients with metastatic CRC. The patients were treated with different types of chemotherapy in different studies. The 25 % survival for CRC patients with isolated PM was 24 months [3]. We therefore defined LTS for CRC-PM patients as survival of at least 24 months.

### Subgroup analysis of PIPAC-OPC1 and PIPAC-OPC2

**Figure 1:**
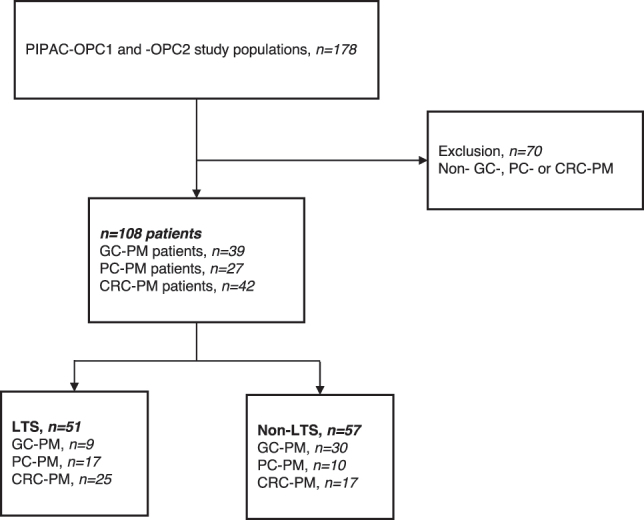
Flow chart of patient inclusion. CRC-PM, peritoneal metastasis (PM) from colorectal cancer; GC-PM, PM from gastric cancer; LTS, long-term survival; OPC, Odense PIPAC Center; PC-PM, PM from pancreatic cancer; PIPAC, pressurized intraperitoneal aerosol chemotherapy.

**Figure 2:**
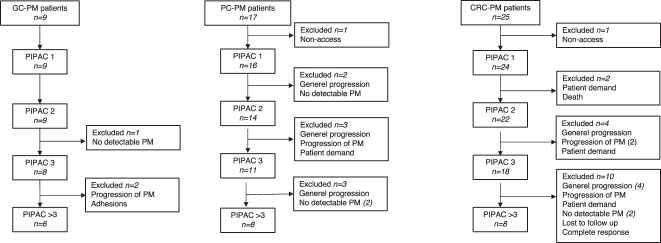
Number of PIPACs, and reasons for discontinued treatment patients with long-term survival. CRC-PM, peritoneal metastasis from colorectal cancer; GC-PM, PM from gastric cancer; LTS, long-term survival; PC-PM, PM from pancreatic cancer; PIPAC, pressurized intraperitoneal aerosol chemotherapy; PM, peritoneal metastasis.

A total of 108 patients with GC-PM, PC-PM, or CRC-PM were treated with PIPAC or ePIPAC, and 51 patients (47.2 %) reached the definition of LTS (nine GC, 17 PC and 25 CRC) (Figure 1). Two hundred two PIPAC procedures were planned for the LTS patients with a median of three PIPAC procedures per patient (range 1–9) (Figure 2). Two, one and five LTS patients with GC-PM, PC-PM and CRC-PM were alive at the date of data extraction.

**Figure 3:**
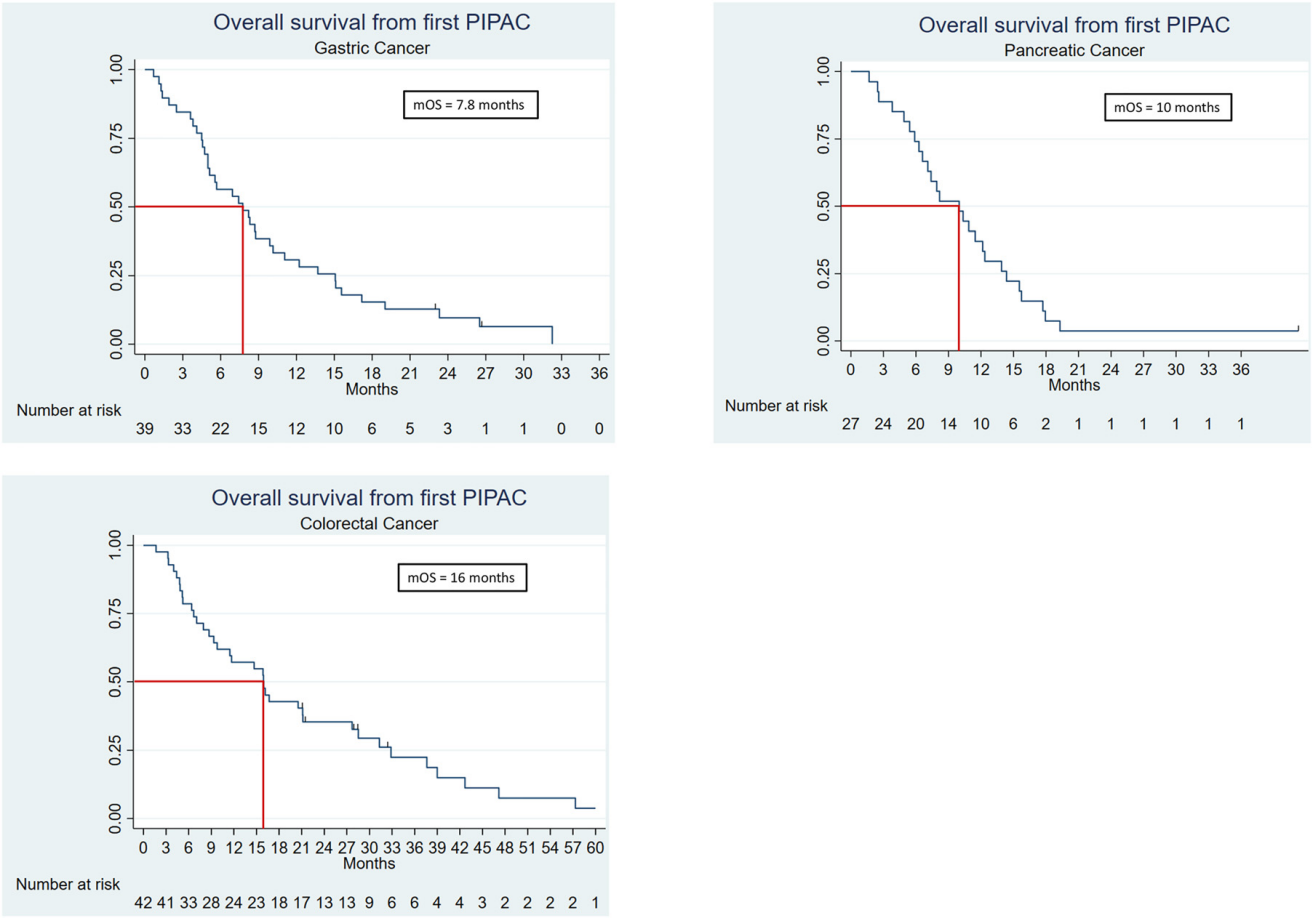
Overall survival from PIPAC 1 in patients with peritoneal metastasis from gastric, pancreatic, and colorectal cancer. mOS, median overall survival; PIPAC, pressurized intraperitoneal aerosol chemotherapy.

Baseline characteristics of patients are summarized in Table 1. All LTS patients but one received palliative chemotherapy prior to PIPAC 1, whereas 19 (37.3 %) LTS patients and 27 (47.4 %) non-LTS patients received bidirectional treatment. The median time from diagnosis of primary tumor to PIPAC 1 was longer in LTS compared to non-LTS PC-PM and CRC-PM patients. For all three origins of cancer, the time from diagnosis of PM to PIPAC 1 was longer among LTS compared to non-LTS patients. The mOS from PIPAC 1 for GC-PM, PC-PM, and CRC-PM LTS was 23.3, 12.4, and 28.5 months. For non-LTS, the mOS from PIPAC 1 for GC-PM, PC-PM, and CRC-PM LTS was 5.6, 5.4, and 7.1 months. Survival by primary tumor for the entire study population is shown in Figure 3.

**Table 1:** Baseline characteristics of patients with peritoneal metastasis by primary tumor and survival.

Cancer origin	Gastric cancer (GC)		Pancreatic cancer (PC)		Colorectal cancer (CRC)	
	LTS	Non-LTS	LTS	Non-LTS	LTS	Non-LTS
Number of patients	9	30	17	10	25	17
Age, years, median (range)	64 (44–76)	61 (31–79)	61 (46–72)	67 (48–72)	61 (39–76)	69 (47–80)
Gender, female/male	3/6	21/9	7/10	5/5	9/16	10/7
Performance status, n (%)						
ECOG 0	5 (56)	11 (37)	10 (59)	1 (10)	12 (48)	6 (35)
ECOG 1	4 (44)	19 (63)	7 (41)	9 (90)	11 (44)	10 (59)
ECOG 2	0	0	0	0	2 (8)	1 (6)
Extraperitoneal disease, n (%)	2 (22)	5 (17)	0	1 (10)	3 (12)	4 (24)
Resected primary tumor, n (%)	1 (11)	8 (27)	5 (29)	2 (20)	19 (76)	14 (82)
CRS+HIPEC, n (%)	0	0	0	0	3 (12)	0
Chemotherapy						
Neoadjuvant, n (%)	1 (11)	9 (30)	0	0	5 (20)	2 (12)
Adjuvant, n (%)	1 (11)	5 (17)	4 (24)	1 (10)	12 (48)	8 (47)
Palliative, n (%)	9 (100)	26 (87)	17 (100)	10 (100)	24 (96)	15 (88)
None, n (%)	0	0	0	0	0	1 (6)
Lines of prior palliative chemotherapy, median (range)	1 (1–3)	1 (1–3)	1 (1–3)	1 (1–2)	1 (1–3)	1 (1–2)
Bidirectional chemotherapy, n (%)	6 (67)	16 (53)	8 (47)	5 (50)	5 (20)	6 (35)
Time from diagnosis of primary tumor to PIPAC 1, months, median (range)^a^	6.5 (3.6–45.5)	6.7 (3.1–24.5)	9.4 (5.5–42.1)	8.4 (4–39.5)	29.4 (8–159)	15.9 (2.4–66.9)
Time from diagnosis of PM to PIPAC 1, months, median (range)	5.1 (2.7–21.7)	4.5 (0.5–14.8)	8.9 (0–34.3)	6.9 (1.2–8.5)	10.3 (0.7–37.4)	7.1 (1.1–15.6)

^a^Data from two patients were excluded since they started PIPAC before final diagnosis of primary tumor. CRC, colorectal cancer; CRS+HIPEC, cytoreductive surgery and heated intraperitoneal chemotherapy; ECOG, Eastern Cooperative Oncology Group; GC, gastric cancer; LTS, long-term survival; n, number of patients; PC, pancreatic cancer; PIPAC, pressurized intraperitoneal aerosol chemotherapy; PM, peritoneal metastasis.

### Response evaluation

#### Histologic response

A total of 41 LTS patients (93.2 %) had a histology-based response (Table 2). Among all LTS patients, 32 (62.8 %) had a mean PRGS ≤2 at PIPAC 1. A total of 28 non-LTS patients (71.8 %) had a histology-based response. Of all non-LTS patients 15 (26.3 %) had a mean PRGS ≤2 at PIPAC 1.

**Table 2:** Number of PIPAC procedures and response evaluation at PIPAC 3.

Cancer origin	Gastric (GC)		Pancreatic (PC)		Colorectal (CRC)	
	LTS	Non-LTS	LTS	Non-LTS	LTS	Non-LTS
Number of patients	9	30	17	10	25	17
PIPAC procedures, median (range)	5 (2–9)	3 (1–7)	3 (1–9)	2 (1–4)	3 (1–9)	1 (1–4)
Patients with 3 or more PIPAC, n (%)	8 (89)	18 (60)	11 (65)	4 (40)	20 (80)	6 (35)
ePIPAC procedures	4	18	3	0	3	6

Histologic response according to PRGS						

Response, n (%)	8 (89)	16 (70)	11 (85)	5 (63)	22 (100)	7 (88)

Cytologic response						

Response, n (%)	1 (14)	2 (10)	0	0	7 (35)	5 (63)
Progression, n (%)	0	7 (35)	3 (21)	0	2 (10)	1 (13)
No change (positive), n (%)	2 (29)	10 (50)	6 (43)	6 (86)	3 (15)	2 (25)
No change (negative), n (%)	4 (57)	1 (5)	5 (36)	1 (14)	8 (40)	0

CRC, colorectal cancer; GC, gastric cancer; LTS, long-term survival; n, number of patients; PC, pancreatic ductal cancer; PIPAC, pressurized intraperitoneal aerosol chemotherapy; PRGS, peritoneal regression grading score.

One PC-PM LTS patient received three PIPAC treatments without peritoneal biopsies and was therefore excluded from the analysis. Information regarding mean PRGS was obtained for all other LTS and all non-LTS patients.

#### Cytologic response

Both response and progression in cytology were observed among the LTS patients (Table 2). The highest occurrence of response was observed among the CRC-PM patients. Negative cytology at baseline and at PIPAC 3 was observed more frequently in LTS compared to non-LTS, for all types of primaries (Table 2). There was no information on cytology in two GC-PM and two CRC-PM LTS patients.

## Discussion

This is, to the best of our knowledge, the first attempt to define long-term survival in patients with GC- (21 months), PC- (15 months) or CRC-PM (24 months). It was based on data from randomized controlled trials or systematic reviews with meta-analysis [3, 9, 38], [39], [40], [41]. We used the definition to investigate if patients with PM reached LTS after a multimodal treatment strategy including systemic chemotherapy and PIPAC. Based on data from the prospective PIPAC-OPC1 and -OPC2 studies of 108 patients with PM from gastrointestinal cancers, we found that 47.2 % patients reached LTS according to these definitions: 23.1 % in GC-PM, 63.0 % in PC-PM and 60.0 % in CRC-PM patients, and most showed histological response.

As patients with PM are known to have a poor prognosis, it is noteworthy that almost half of our cohort reached LTS. The observed mOS from PIPAC 1 of 23.3 months for GC-PM and 28.5 months for CRC-PM are also higher than previously reported [16, 18], [19], [20, 27, 28, 30, 36]. Of course, this is expected since the previously reported mOS is more representative of complete study populations, as opposed to the patients who live the longest. The mOS of 12.4 months for PC-PM LTS patients is also interesting compared to previous studies, but heterogeneity and size of respective study populations does not allow further computation or comparison [18, 32], [33], [34]. We must also recognize that even if the patients achieved our definition of LTS, the survival curves show that only a minority of patients were alive at the date of data extraction. This outlines the continuously fatal outcome of patients with PM. Still, two important and clinically relevant parameters should be highlighted when discussing the selection of patients and assessment of treatment response. First, the selection of patients based on the Eastern Cooperative Oncology Group (ECOG) performance status (PS) is not uniform. Some studies included patients with a PS of 2, while others did not use or report the performance status at all [18], [19], [20, 27, 32], [33], [34]. In PIPAC-OPC2, only patients with a PS of 0–1 were included. Thus 96 % of our LTS had a PS of 0–1 which potentially imposed a selection bias since patients with a good PS arguably live longer and tolerate more palliative treatment than patients with a PS >1. On the other hand, a PS of 0–1 is a standard criterion in most large scale RCTs in the palliative setting. Second, we observed a high number of LTS patients with response to PIPAC according to PRGS. At PIPAC 3, 41 (93.2 %) of the LTS patients and 28 (71.8 %) non-LTS patients responded to treatment with a PRGS ≤2 or an absolute decrease of more than 1. Of note, 51 (47.2 %) LTS and non-LTS patients had a mean PRGS ≤2 at PIPAC 1, which presumably represents a response to previous systemic treatment. However, it is still possible that PIPAC treatment may have contributed to maintain a low PM burden in patients with a PRGS ≤2 at PIPAC 1 and PIPAC 3. The prognostic value of PRGS has been debated, but the PIPAC-OPC2 study of 110 patients with different primary tumors showed that a complete or major histological response to PIPAC treatment was achieved in 61 % of patients who had three PIPACs. Importantly, this finding was the only independent prognostic factor in multivariate analysis [37]. In comparison, Benzerdjeb et al. showed prognostic value of combining peritoneal cytology and PRGS in patients with PM, and recently Baake et al. showed both a predictive as well as a prognostic value of PRGS before and during PIPAC [46, 47]. Data from the present study were too small to evaluate a potential effect on survival following PIPAC eradication of (cytological detected) intraperitoneal malignant cells.

The retrospective design and heterogeneous study population, which was pooled from two prospective studies, impose limitations and should be considered shortcomings of this study. The subgroup analyses based on primary tumor (GC, PC, CRC) should be considered a strength that attempted to reduce this heterogeneity. Different interventions including the use of ePIPAC in some of our patients, hamper the assessment of causality between treatment and effect. On the other hand, only five LTS patients (10 %) received ePIPAC, and four of these patients had both ePIPAC and PIPAC. The median time from diagnosis of PM to PIPAC was longer among the patients with LTS compared to non-LTS. This may indicate that these patients received palliative treatment for a longer time period and generally responded better to systemic treatment. Therefore, it is not known if and to what extent the survival data are affected by selection bias, the PIPAC treatment per se or the prior (or subsequent) systemic treatment. Most likely, the survival is achieved through a combination of all these important elements. Odense PIPAC Center is the national center for PIPAC in Denmark, and patients are referred from the oncological departments from the entire country. Therefore, data on previous types and lines of chemotherapy including toxicity and response data may be scarce, which potentially introduces missing data in baseline characteristics of the study population. Six patients were discontinued after PIPAC 1, 2, or 3 because they had no detectable PM at laparoscopy. All of these had severe adhesions, which made a complete laparoscopic evaluation of the peritoneal cavity impossible. It is not known if PIPAC had an impact on survival in these patients. A subset of patients in both groups had bidirectional treatment, but there were no predefined criteria that described how to select patients for this treatment instead of PIPAC as monotherapy, which is a limitation [48]. Of note, this decision was according to everyday practice taken on the dedicated multi-disciplinary team conference. Interestingly, according to PRGS, data did not reveal better effect in bidirectional treated patients compared to PIPAC monotherapy. Survival data in this study must be interpreted with caution since information regarding the potential reintroduction of systemic treatment after PIPAC is missing. These data are also lacking in most PIPAC studies, an important topic which should be addressed in future studies. The study is strengthened through the use of data from two prospective studies, and of one pathologist with special interest in peritoneal surface malignancies. This eliminated inter-observer disagreement regarding histology and cytology based response assessment [43]. Of note, peritoneal biopsies were analysed by the use of up-front immunohistochemistry, which is pivotal in this setting.

This study suggests a definition of LTS from the date of diagnosis of PM or metastatic disease. Our LTS definitions were based on RCTs and systematic reviews investigating metastatic disease in general. Future large scale randomized studies in well-defined study populations must be conducted to make more strict definitions, but also to elucidate the survival benefit of both systemic treatment and PIPAC in patients with PM. Future studies must also investigate the optimal timing of introducing PIPAC in the course of therapy in patients with PM. As shown by our data, PIPAC is usually not used before the patients have been treated for several months with systemic chemotherapy. Based on the large number of patients with histology based response to PIPAC, survival might be improved by introducing PIPAC in the first line of palliative treatment, which must be a priority in the design of future studies.

In conclusion, this study highlights that a substantial proportion of patients with PM from GC, PC, and CRC can achieve long-term survival by means of both systemic treatment and PIPAC. It seems possible to identify candidates and responders by using the histology based PRGS. The findings of this study are intriguing, but large randomized trials are needed to define efficacy, and to decode the true causality of long-term survival.
